# Type 2 diabetes: prevalence and associated factors in a Brazilian community – the Bambuí health and aging study

**DOI:** 10.1590/S1516-31802005000200007

**Published:** 2005-03-02

**Authors:** Valéria Maria de Azeredo Passos, Sandhi Maria Barreto, Leonardo Maurício Diniz, Maria Fernanda Lima-Costa

**Keywords:** Diabetes mellitus, Glucose intolerance, Aging, Preventive medicine, Body mass index, Diabetes Mellitus, Envelhecimento, Medicina preventiva, Intolerância a glucose, Índice de massa corporal

## Abstract

**CONTEXT AND OBJECTIVE:**

Diabetes is an increasing cause of death in developing countries. Our objective was to describe the prevalence and clinical factors associated with diabetes and impaired fasting glycemia among adults (18-59 years) and elderly adults (60+ years).

**DESIGN AND SETTING:**

Population based, cross-sectional study in Bambuí, Brazil.

**METHODS:**

816 adult and 1,494 elderly participants were interviewed; weight, height and blood pressure measured; and blood samples collected. Diabetes was defined as plasma fasting glucose ≥ 126 mg/dl and/or use of hypoglycemic agents; impaired fasting glycemia as glycemia of 110-125 mg/dl. Associations were investigated using multinomial logistical regression (reference: fasting glycemia ≤ 109 mg/dl).

**RESULTS:**

Among the elderly, 218 (14.59%) presented diabetes and 199 (13.32%) impaired fasting glycemia, whereas adult prevalences were 2.33% and 5.64%. After multinomial analysis, diabetes remained associated, for adults, with increased waist-to-hip ratio and total cholesterol ≥ 240 mg/dl; for elderly adults, with family history of diabetes, body-mass index of 25-29 kg/m^2^, body-mass index ≥ 30 kg/m^2^, increased waist-to-hip ratio, low HDL-cholesterol triglyceridemia of 200-499 mg/dl and triglyceridemia ≥ 500 mg/dl. Among adults, impaired fasting glycemia remained associated negatively with male sex and positively with ages of 40-59 years, physical inactivity and increased waist-to-hip ratio; among the elderly, with alcohol consumption, overweight, obesity and triglycerides > 200 mg/dl.

**CONCLUSIONS:**

The results reinforce the importance of interventions to reduce physical inactivity, alcohol consumption, obesity and dyslipidemia, so as to prevent increasing incidence of diabetes.

## RESUMO

**CONTEXTO E OBJETIVO:**

O diabetes é uma causa crescente de mortalidade nos países em desenvolvimento. O objetivo foi descrever a prevalência e fatores clínicos associados ao diabetes e à glicemia de jejum alterada em adultos (18-59 anos) e idosos (60+ anos).

**TIPO DE ESTUDO E LOCAL:**

Transversal de base populacional em Bambuí, Brasil.

**MÉTODOS:**

Foram entrevistados 816 adultos e 1.494 idosos; foram feitas medidas antropométricas, da pressão arterial e exames laboratoriais. O diabetes foi definido como glicemia de jejum ≥ 126 mg/dl e/ou uso de drogas hipoglicemiantes. Glicemia de jejum alterada foi definida como glicemia 110-125 mg/dl. Associações foram investigadas utilizando-se a regressão logística multinomial (referência: glicemia de jejum ≤ 109 mg/dl).

**RESULTADOS:**

Entre os idosos, 218 (14,59%) apresentavam diabetes e 99 (13,32%) glicemia de jejum alterada, enquanto foram 2,33% e 5,64% nos adultos. Após análise multinomial, o diabetes manteve-se associado nos adultos ao aumento da razão cintura-quadril e colesterol total ≥ 240 mg/dl; nos idosos, à história familiar da doença, índice de massa corporal 25-29 kg/m^2^, índice de massa corporal ≥ 30 kg/m^2^, trigliceridemia 200-499 mg/dl e trigliceridemia ≥ 500 mg/dl. Nos adultos, glicemia de jejum alterada manteve-se associada negativamente ao sexo masculino e positivamente à idade 40-59 anos, inatividade física e razão cintura-quadril elevada; nos idosos, ao uso de bebidas alcoólicas, sobrepeso, obesidade e trigliceridemia > 200 mg/dl.

**CONCLUSÕES:**

Nossos resultados reforçam importância de intervenções para redução da inatividade física, consumo de álcool, obesidade e dislipidemias, visando deter a crescente incidência do diabetes.

## INTRODUCTION

Diabetes mellitus type 2 is one of the ten leading causes of death in the world. Its incidence is increasing, especially in developing countries. A 35% increase in the prevalence of diabetes is expected between 1995 and 2025 among those aged 20 years of age or over. Despite the higher prevalence of diabetes in developed countries, the absolute increase will more heavily affect developing countries, where the majority of the world's population lives. Developing countries will also suffer as a consequence of the rapid aging process that their populations will undergo, since the prevalence of diabetes increases with age. It has thus been estimated that the number of adults with diabetes will increase from 4.9 million in 1995 to approximately 11.6 million in 2025.^1^ A multicenter study on the prevalence of diabetes and impaired glucose tolerance that was conducted among adults (30-69 years of age) in nine Brazilian cities showed values of 7.6% and 7.8%, respectively.^2^

Very little is known about the health conditions of Brazilians living in small cities. In Brazil, 72% of the municipalities have less than 20,000 inhabitants, corresponding to 19% (27 million) of the total Brazilian population. There is a common belief that the increase in the prevalence of risk factors for chronic degenerative diseases is a public health problem mostly for large urban centers. However, this may not be true. Results from studies undertaken in Bambuí, a small city in the state of Minas Gerais, have found a high prevalence of obesity,^3^ hypertension,^4^ intermittent claudication^5^ and coronary heart disease.^6^ Bambuí is a typical example of the coexistence of two epidemiological profiles of morbidity: high prevalence of risk factors for chronic and degenerative diseases with elevated prevalence of infection by *Trypanosoma cruzi,* especially among elderly adults.^7^

### Objectives

We present here the results of a population-based study regarding the prevalence and associated factors of diabetes and impaired fasting glycemia among adults and elderly adults living in the community of Bambuí.

## METHODS

We analyzed the baseline data of the Bambuí Health and Aging Study (BHAS), a prospective study for which the methodology has already been published.^8^ Bambuí now has about 22,000 inhabitants, 80% of them in the urban area of the municipality.^9^

The Ethics Committee of Fundação Os-waldo Cruz (Fiocruz) approved this study. Out of a total of 1,742 residents in the municipality aged 60 years or over in 1997, 1,494 (85.7%) were interviewed and examined. Adults aged 18-59 years were selected through a non-replaceable simple probabilistic sample out of a total population within this age range of 8,899. The parameters used to calculate the sample size (1,020/8,899) were prevalence equal to 50%, confidence interval = 0.95, losses = 0.20 and precision = 0.30. Eighty percent of the adults selected (816/1,020) were interviewed and examined. The distribution of older and younger participants was similar to the original population, with regard to gender, age, marital status, monthly family income and education.^8^

After informed written consent had been obtained, previously trained assistants undertook an interview in the participants’ homes, using a pre-coded questionnaire. The following variables from the baseline interview were included in the present study: a) sociodemographic characteristics (age, gender, education and monthly income); b) lifestyle habits (physical activity, current smoking status, use of alcohol); c) history of selected diseases (angina, infarction, stroke, intermittent claudication); d) family history of diabetes among first-degree relatives and of cardiovascular diseases before 50 years of age; and f) health service indicators. The use of medicines was verified from the labeling of medicines being used at the time of the interview.

Physical examinations and blood tests were undertaken at an outpatient clinic or at home, when the subject was in a poor state of health. Specially trained health technicians performed anthropometric measurements using standard equipment, on barefoot individuals wearing light clothing. Weight (kg) and height (cm) were used to obtain the body mass index (weight/height^2^). Obesity was defined as body mass index ≥ 30 kg/m^2^ and overweight as body mass index of 25-29 kg/m^2^. The waist circumference (cm) and hip circumference (cm) were measured to obtain the waist-to-hip ratio, and normal values were considered to be up to 1.0 for men and 0.95 for women.^3^ The waist circumference was measured using a non-elastic tape measure, at the smallest diameter between the iliac crest and outer face of the last rib. The hip circumference was measured using a non-elastic tape measure, at the point of greatest perimeter between the hips and buttocks.

Physical inactivity in daily life was defined as walking or doing other exercise less than once a week and/or by self-perception of being sedentary.^8^ Myocardial infarction was defined through a medical report and angina was defined from a history of chest pain after exertion lasting for up to 10 minutes that disappeared with rest or the use of nitrates.^10^ Intermittent claudication was defined when the interviewer reported pain in the calf while walking that was not associated with the standing or sitting position, lasted for up to 10 minutes and disappeared with pace reduction or interruption of gait.^10^

Blood pressure was measured with the patient in the sitting position, after at least five minutes of rest, and without caffeine or tobacco having been used for at least the previous 30 minutes. Three measurements were taken at intervals of two minutes; the first of these was discarded and the blood pressure obtained from the mean of the latter two measurements. Hypertension was defined as blood pressure ≥ 140/90 mmHg and/or current use of antihypertensive drugs.^11^

Blood samples were collected with 12-hour fasting for biochemical analysis (glucose, creati-nine, urea, uric acid, total and partial cholesterol and triglycerides). For glucose tests, blood was collected in fluorinated tubes and plasma was immediately separated and kept refrigerated at 4° C for up to 48 hours. Plasma glucose was determined by the glucose-oxidase method. Diabetes was defined as a fasting glucose level of 126 mg/dl or more, or if current use of insulin or oral hypoglycemic agents was reported. Impaired fasting glycemia was defined as fasting glucose of between 110 and 125 mg/dl.^12^ Triglyceride levels were grouped into three categories (< 200, 200-499 and ≥ 500 mg/dl).^13^ Renal dysfunction was defined as plasma creatinine levels ≥ 1.3 mg/dl for men or ≥ 1.2 mg/dl for women.^14^

***Statistical analysis.*** The data were analyzed using the Stata (StataCorp, 2003) software.^15^ The prevalences were determined separately for adults and elderly adults and the estimates of prevalence in the overall population were made using weighting procedures to correct for the effects of differences in sample size between the adult and elderly adult populations. The magnitude of the association between the variables was determined by means of odds ratios (OR) and confidence intervals (CI), calculated using the Woolf method.^16^

Because the dependent variable was composed of three categories (glucose < 109 mg/dl, glucose of 110-125 mg/dl and glucose > 126 mg/dl), we employed multinomial logistic regression techniques to estimate the association of glucose levels with independent variables. The glucose level of up to 109 mg/dl was taken as the reference category. The following criteria guided the inclusion of variables in the logistic model: a) the existence of an association between the independent and dependent variables at a level below 20% (p < 0.20); and b) variables that, even though not presenting an association at this level could be potential confounding factors (gender and age, for instance). In the final model, variables were regarded as statistically associated with the dependent variables when p values were equal to or less than 0.05.

## RESULTS

Table 1 shows the prevalences of the principal clinical characteristics of the studied population. The elderly adult group showed higher prevalence rates for physical inactivity, current smokers, hypertension, total cholesterol and triglycerides levels than did the adult group. No difference was found in relation to body mass index between adults and elderly adults, but the waist-to-hip ratio was significantly greater among the elderly adults and among women of both age groups.

**Table 1:** Clinical and laboratory characteristics of participants in the Bambuí Health and Aging Study, 1997

Characteristic	Adults (18-59 years of age)		Elderly adults (60+ years of age)	
	Men	Women	Men	Women
Age, years (mean SD)	35.9 ± 12.1	36.6 ± 11.4	68.8 ± 7.1	69.3 ± 7.2
Current smoker (%)*	38.8	23.9	30.6	9.9
Physical inactivity (%)*	10.3	13.7	28.3	38.7
Systolic blood pressure, (mmHg mean SD)	118.3 ± 17.3	112.6 ± 18.9	137.7 ± 22.9	137.1 ± 22.4
Diastolic blood pressure, (mmHg mean SD)	77.4 ± 11.4	74.4 ± 12.8	84.9 ± 13.2	82.4 ± 12.2
Hypertension (%)*	18.1	22.1	59.7	59.8
Body mass index, (kg/m^2^ mean SD)	24.4 ± 3.7	25.23 ± 5.2	23.9 ± 4.1	25.8 ± 5.2
Increased waist-to-hip ratio (%)*	14.96	31.29	39.01	88.67
Creatinine, (mg/dl mean SD)	0.9 ± 0.1	0.7 ± 0.1	1.0 ± 0.4	0.8 ± 0.2
Glucose, (mg/dl mean SD)	96.3 ± 22.6	103.1 ± 36.7	107.9 ± 45.4	109.0 ± 41.9
Uric acid, (mg/dl mean SD)	5.7 ± 1.6	4.1 ± 1.4	5.8 ± 1.7	5.1 ± 1.6
Cholesterol, (mg/dl mean SD)*	186.6 ± 41.4	186.1 ± 41.8	219.7 ± 46.0	241.8 ± 49.1
HDL-cholesterol, (mg/dl mean SD)	49.4 ± 15.3	53.0 ± 14.7	46.9 ± 15.7	50.6 ± 14.4
Triglycerides, (mg/dl mean SD)*	142.6 ± 115.2	123.5 ± 84.0	135.7 ± 89.9	168.9 ± 107.3

* *Statistically significant differences at the level of p < 0.01.*

*SD = standard deviation; HDL = high-density lipoprotein.*

Among the 816 adults, 46 (5.64%) presented glucose levels compatible with impaired fasting glycemia and 19 (2.33%) with diabetes; among these, only six used specific medicines. Univariate analysis among the adults (Tables 2 and 3) showed that elevated glucose levels were statistically associated with gender, age group, skin color, educational level, head-of-family status, physical activity, high body mass index, increased waist-to-hip ratio, presence of hypertension, previous diagnosis of myocardial infarction, previous stroke, hypercholesterolemia, low HDL-C (high-density lipoprotein cholesterol) and hypertriglyceridemia. All these variables were included in the logistic model.

**Table 2:** Univariate analysis for glycemic levels, sociodemographic variables and lifestyle habits in the adult population (18-59 years of age) of Bambuí, 1997

Variable	Fasting glucose (mg/dl)		Diabetes	Chi-squared
	≤ 109	110-125		p
	n = 751	n= 46	n = 19	
**Gender**				
Female	431	16	11	9.1155
Male	323	30	8	0.010
**Age group (years)**				
18-29	264	6	0	
30-39	211	7	2	40.2298
40-49	164	15	7	0.000
50-59	112	18	10	
**Skin color**				
White	394	30	6	6.3085
Non-white	357	16	13	0.043
**Education (years)**				
0	47	2	4	
1-3	118	17	5	23.0123
4-7	293	15	6	0.001
8+	293	12	4	
**Head of family**				
No	439	17	10	8.381
Yes	312	29	9	0.015
**Daily physical activity**				
Sedentary	668	33	15	13.3378
Light/moderate	83	13	4	0.001

**Table 3:** Univariate analysis for glycemic levels and clinical/laboratory variables in the adult population (18-59 years of age) of Bambuí, 1997

Variable	Fasting glucose (mg/dl)		Diabetes	Chi-squared
	≤ 109	110-125		p
	n = 751	n= 46	n = 19	
**Body mass index (kg/m^2^)**				
< 25	430	18	5	
25-29.9	18	18	7	15.8156
≥ 30	5	10	6	0.003
**Hypertension**				
No	636	31	10	21.84
Yes	115	15	9	0.000
**Myocardial infarction**				
No	741	44	18	9.6432
Yes	5	2	1	0.008
**Stroke**				
No	741	42	19	15.8293
Yes	9	4	0	0.000
**Cholesterol (mg/dl)**				
< 200	508	20	5	
200-239	179	15	9	29.5851
≥ 240	64	11	5	0.000
**Low HDL cholesterol**				
No	320	24	13	6.42
Yes	431	22	6	0.040
**Triglycerides (mg/dl)**				
< 200	658	28	7	
> 200-499	86	15	3	40.4641
> 500	7	3	1	0.000

*HDL = high-density lipoprotein.*

Among the adults, after adjustment by the multinomial analysis, impaired fasting glycemia remained negatively associated with male gender and positively associated with ages between 40-49 years, 50-59 years, physical inactivity and increased waist-to-hip ratio. Diabetes remained associated with increased waist-to-hip ratio and high cholesterol levels. The associations between glucose levels and body mass index disappeared when adjusted for waist-to-hip ratio (Table 4).

**Table 4:** Multinomial analysis for glycemic levels and variables associated with the adult population (18-59 years of age) of Bambuí, 1997

Variable	Fasting glucose		OR	Diabetes	OR
	≤ 109	110-125	(95% CI)	n = 19	(95% CI)
	n = 75	n = 46			
**Gender**					
Female	431	16	1.00	11	1.00
Male	323	30	**0.35 (0.17-0.72)**	8	0.57 (0.21-1.60)
**Age group**					
18-29 years	264	6	1.00	0	1.00
30-39 years	211	7	1.36 (0.44-4.18)	2	1.03 (0.14-7.60)
40-49 years	164	15	**3.80 (1.28-11.29)**	7	3.86 (0.76-19.78)
50-59 years	112	18	**5.93 (2.01-17.51)**	10	4.63 (0.88-24.40)
**Physical activity**					
Yes	83	13	1.00	4	1.00
No	668	33	**3.13 (1.41-6.93)**	15	1.10 (0.29-4.59)
**Increased waist-to-hip ratio**					
No	685	38	1.00	12	1.00
Yes	38	7	**2.92 (1.02-8.39)**	7	**4.99 (1.47-17.0)**
**Total cholesterol (mg/dl)**					
< 240	687	35	1.00	14	1.00
≥ 240	64	11	1.13 (0.45-2.87)	5	**3.22 (1.14-9.12)**

*OR = odds ratio; CI = confidence interval.*

* *The variables that presented a statistical association or some confounding potential (gender and age) were maintained. The OR values were simultaneously adjusted for all the variables present in the table.*

Among the elderly adults, 199 (13.32%) were classified as presenting impaired fasting glycemia and 218 (14.59%) as diabetic, of whom 100 were taking drugs. In the univariate analysis (Tables 5 and 6), diabetes was statistically associated with lower levels of education and income, smoking habit, use of alcoholic drinks, high body mass index, presence of hypertension, family history of diabetes and hypertriglyceridemia. All these variables were included in the multinomial logistical model.

**Table 5:** Univariate analysis for glycemic levels, sociodemographic variables and lifestyle habits in the elderly adult population (60+ years of age) of Bambuí, 1997

Variable	Fasting glucose (mg/dl)		Diabetes	Chi-squared
	≤ 109	110-125		p
	n = 1077	n= 199	n = 218	
**Gender**				
Female	647	118	143	2.5420
Male	430	81	75	0.281
**Age group (years)**				
60-69	622	129	132	
70-79	336	53	71	6.5833
80+	119	17	15	0.160
**Education (years)**				
0	360	56	52	
1 - 3	342	68	76	13.2507
4 - 7	278	62	73	0.039
8+	90	13	17	
Income (minimum salaries)*				
< 2	706	126	128	
2-3.9	146	34	33	13.7552
4-5.9	64	11	12	0.032
6+	66	9	26	
**Smoking habit**				
No	643	125	127	
Ex-smoker	216	24	29	14.5696
Smoker	228	50	62	0.006
**Use of alcoholic drinks (frequency)**				
Never	502	78	107	
Less than once a week	434	85	95	12.5757
At least once a week	107	36	16	0.014

* *R$120.00 was the value of one minimum salary in Brazil in 1997 and it was R$ 260.00 in 2004 (around U$100).*

**Table 6:** Univariate analysis for glycemic levels and clinical/laboratory variables in the elderly adult population (60+ years of age) of Bambuí, 1997

Variable	Fasting glucose (mg/dl)		Diabetes	Chi-squared
	≤ 109	110-125		p
	n = 1077	n= 199	n = 218	
**Body mass index (kg/m^2^)**				
< 25	595	77	73	76.7723
25-29.9	346	79	87	0.000
≥ 30	91	41	53	
**Hypertension**				
No	447	66	63	14.9737
Yes	630	133	155	0.001
**Family history of diabetes**				
No	271	64	97	36.1150
Yes	767	133	110	0.000
**Triglycerides (mg/dl)**				
< 200	906	143	142	
200-499	167	52	64	71.0618
> 500	4	4	12	0.0000

Table 7 shows the results of the multi-nomial analysis among the elderly adults. Impaired fasting glycemia remained associated with the use of alcoholic drinks, overweight, obesity, and triglyceride levels of over 200 mg/dl. Diabetes remained associated with a positive family history of diabetes, overweight, obesity, increased waist-to-hip ratio, triglycerides of over 200 mg/dl and low HDL-C.

**Table 7:** Multinomial analysis for glycemic levels and variables associated with the elderly adult population (60+ years of age) of Bambuí, 1997

Variable	Fasting glucose (mg/dl)		OR	Diabetes	OR
	≤ 109	110-125			
	n = 75	n = 46	(95% CI)	n = 19	(95% CI)
**Gender**					
Female	647	118	1.0	143	1.00
Male	430	81	1.04 (0.66-1.64)	75	1.10 (0.67-1.77)
**Age group (years)**					
60-69	622	129	1.00	132	1.00
70-79	336	53	1.06 (0.56-2.01)	71	1.14 (0.56-2.27)
80+	119	17	0.93 (0.47-1.81)	15	1.34 (0.65-2.73)
**Family history of diabetes**					
No	271	64	1.00	97	1.00
Yes	767	133	1.21 (0.84-1.73)	110	**1.92 (1.36-2.73)**
**Use of alcohol (frequency)**					
Never	502	78	1.00	107	1.00
< 1 once a week	434	85	1.39 (0.94-2.05)	95	1.20 (0.81-1.77)
At least once a week	139	36	**1.82 (1.01-3.30)**	16	0.61 (0275-1.33)
**Body mass index (kg/m^2^)**					
< 25	595	77	1.00	73	1.00
25-29.9	346	79	**1.73 (1.17-2.56)**	87	1.64 (1.10-2.45)
≥ 30	91	41	**3.14 (1.91-5.16)**	53	3.52 (2.18-5.70)
**Increased waist-to-hip ratio**					
No	684	123	1.00	120	1.00
Yes	306	54	0.94 (0.65-1.38)	89	**1.79 (1.25-2.56)**
**Low HDL-C**					
No	504	111	1.00	134	1.00
Yes	573	88	1.01 (0.69-1.46)	84	1.36 (1.00-1.99)
**Triglycerides (mg/dl)**					
< 200	988	170	1.00	170	1.00
200-499	80	25	**1.83 (1.21-2.77)**	34	**1.71 (1.13-2.59)**
≥ 500	9	4	**10.64 (1.84-61.7)**	14	**20.82 (4.15-94.4)**

* *The variables that presented a statistical association or some confounding potential (gender and age) were maintained. The OR values were simultaneously adjusted for all the variables present in the table.*

*OR = odds ratio; CI = confidence interval; HDL-C = high-density lipoprotein cholesterol.*

## DISCUSSION

Our results have confirmed the trend of increasing prevalence of diabetes and impaired fasting glycemia with aging and also show prevalence levels very close to those found in Brazilian metropolitan areas and other developed countries.^2^ The prevalence of diabetes in Bambuí was similar for the two genders and increased with age, with the same distribution pattern as observed in Brazilian urban populations in 1992.^2^ In the present study, lower education did not remain associated with any of the events considered in the multinomial analysis. These findings differ from studies of urban Bolivians and Jamaicans, which showed higher prevalence of diabetes among people with lower educational levels.^17^

In the present study, impaired fasting glycemia and diabetes were associated with non-modifiable risk factors: gender and age among the adults and a positive family history among the elderly adults. Otherwise, high glucose levels were also associated with alcohol use, physical inactivity, non-localized and central obesity, high total cholesterol, low HDL-cholesterol and hypertriglyceridemia.

Type 2 diabetes results from an interaction between genetic and environmental factors. The rapidly changing incidence rates all over the world, however, suggest a particularly important role for the latter as a potential means for stemming the tide of the global epidemic of the disease. The most dramatic increases in type 2 diabetes are occurring in societies in which there have been major changes in the type of diet consumed, reductions in physical activity and increases in overweight and obesity rates.^18^

The type of obesity associated with the incidence of diabetes is a pattern of upper body obesity and visceral fat. In the present study, the association of diabetes with non-localized obesity, characterized by high body-mass index, and with central obesity, defined by increased waist-to-hip ratio, suggests a common lifestyle pattern for this small Brazilian community and highly urbanized Western societies. Among adults, the waist-to-hip ratio was a more powerful determinant of elevated glucose levels than body mass index, as already observed in other studies.^18^

A sedentary lifestyle has also been associated with insulin resistance among non-diabetic individuals, independent of obesity.^19,20^ The regular practice of exercise increases the number of capillaries and muscle fibers, thereby favoring the availability of glucose mediated by insulin from these cells.^21,22^ It has already been demonstrated that even the practice of bouts of exercise stimulates the translocation of GLUT-4 to the plasmatic membrane and increases the transportation of glucose to skeletal muscles.^20^

A single laboratory determination of glycemia level is insufficient to establish a diagnosis for individual cases of diabetes, but it is widely used in population studies for risk estimation and health promotion initiatives,^23^ since early diagnosis of diabetes is essential for preventing secondary complications of the disease. At the time of diagnosis, 9% of patients already have overt cardiovascular problems, 18% retinopathy, 4% nephropathy, 13% neuropathy (absence of two or more reflexes) and 12% the absence of peripheral pulses.^24^

In the present study, we used the criteria recommended by the American Diabetes Association (ADA)^12^ for the diagnosis of diabetes and impaired fasting glycemia. These criteria aim at simplifying the World Health Organization (WHO) diagnostic criteria,^25^ which require fasting glycemia plus another measurement after intake of a glucose solution. The simplicity and practicality of the ADA criteria, which are based on a single fasting plasma measurement, facilitates their use in large-scale preventive interventions for early diagnosis. Among Brazilians with Japanese ancestry aged 40 to 79 years, a comparison of the ADA and WHO criteria showed they had similar sensitivity for the detection of diabetes, but ADA had poorer sensitivity than WHO for the detection of glucose intolerance.^26^ Assuming that the same sensitivities apply to the population of Bambuí, our results underestimate the true prevalence of impaired fasting glycemia.

As Brazil is aging very fast, our results reinforce the importance of early intervention to prevent diabetes, with emphasis on lifestyle modification, especially regarding physical inactivity, prevention of obesity and restriction of alcohol consumption. Most studies and interventions on prevention and health promotion have been conducted among young adults and the middle-aged. However, epidemiological studies show that it is unjustifiable to direct such interventions only to the young and adult populations. The risk factors that influence the development of type 2 diabetes and cardiovascular disease among elderly adults are practically the same as those that apply in middle age, and the potential benefit of preventive measures also extends to older individuals. In fact, even when introduced late in life, these benefits are considerable and substantial, and even more cost-effective among elderly adults than among middle-aged adults, because of the higher risk of cases of disease among elderly adults.^27^

## CONCLUSIONS

Among the adults, the prevalence of diabetes was higher among those with high waist-to-hip ratios and cholesterol levels. Among the elderly adults, the presence of diabetes was associated with an increased waist-to-hip ratio, family history of diabetes, low HDLcholesterol and high triglyceride levels.

Central obesity, expressed by an increased waist-to-hip ratio, was an important factor associated with diabetes in both adults and elderly adults. Our results suggest that waist-to-hip ratio is a better predictor of diabetes than the body mass index.

The results from this study show that diabetes and associated factors are also a problem in a small community in southeastern Brazil. Both modified and non-modified factors for diabetes are present and deserve public healthcare attention so as to prevent future increases in diabetes and its complications in this community.
